# Dietary protein sources and tumoral overexpression of *RhoA*, *VEGF-A* and *VEGFR2* genes among breast cancer patients

**DOI:** 10.1186/s12263-019-0645-7

**Published:** 2019-07-09

**Authors:** Ali Shokri, Saeed Pirouzpanah, Mitra Foroutan-Ghaznavi, Vahid Montazeri, Ashraf Fakhrjou, Hojjatollah Nozad-Charoudeh, Gholamreza Tavoosidana

**Affiliations:** 1 Drug Applied Research Center, Tabriz University of Medical Sciences, Tabriz, Iran; 2 Molecular Medicine Research Center, Tabriz University of Medical Sciences, Tabriz, Iran; 3 Department of Biochemistry and Dietetics, Faculty of Nutrition and Food Sciences, Tabriz University of Medical Sciences, Tabriz, Iran; 4 Department of Clinical Nutrition, Faculty of Nutrition and Food Sciences, Shiraz University of Medical Sciences, Shiraz, Iran; 5 Department of Thoracic Surgery, Faculty of Medicine, Surgery Ward, Nour-Nejat Hospital, Tabriz University of Medical Sciences, Tabriz, Iran; 6 Department of Pathology, Faculty of Medicine, Tabriz University of Medical Sciences, Tabriz, Iran; 7 Stem Cell Research Center, Tabriz University of Medical Sciences, Tabriz, Iran; 8 Department of Molecular Medicine, Faculty of Advanced Technologies in Medicine, Tehran University of Medical Sciences, Tehran, Iran

**Keywords:** Breast cancer, Protein, Metastasis, Angiogenesis, *RhoA*, *VEGF-A*, *VEGFR-2*

## Abstract

**Background:**

High protein intake may promote angiogenesis giving support to the development of metastasis according to the experimental data. However, nutritional epidemiologic evidence is inconsistent with metastasis. Therefore, we aimed to study the association between dietary intake of protein and tumoral expression levels of *Ras homologous gene family member A* (*RhoA*), *vascular endothelial growth factor-A* (*VEGF-A*), and *VEGF receptor-2* (*VEGFR2*) in primary breast cancer (BC) patients.

**Methods:**

Over this consecutive case series, 177 women primary diagnosed with histopathologically confirmed BC in Tabriz (Iran) were enrolled between May 2011 and November 2016. A validated food frequency questionnaire was completed for eligible participants. Fold change in gene expression was measured using quantitative real-time PCR. Principal component factor analysis (PCA) was used to express dietary groups of proteins.

**Results:**

Total protein intake was associated with the expression level of *VEGF-A* in progesterone receptor-positive (PR+: *β* = 0.296, *p* < 0.01) and *VEGFR2* in patients with involvement of axillary lymph node metastasis (ALNM+: *β* = 0.295, *p* < 0.01) when covariates were adjusted. High animal protein intake was correlated with overexpression of *RhoA* in tumors with estrogen receptor-positive (ER+: *β* = 0.230, *p* < 0.05), ALNM+ (*β* = 0.238, *p* < 0.05), and vascular invasion (VI+: *β* = 0.313, *p* < 0.01). Animal protein intake was correlated with the overexpression of *VEGFR2* when tumors were positive for hormonal receptors (ER+: *β* = 0.299, *p* < 0.01; PR+: *β* = 0.296, *p* < 0.01). Based on the PCA outputs, protein provided by whole meat (white and red meat) was associated inversely with *RhoA* expression in ALNM+ (*β* = − 0.253, *p* < 0.05) and premenopausal women (*β* = − 0.285, *p* < 0.01) in adjusted models. Whole meat was correlated with *VEGFR2* overexpression in VI+ (*β* = 0.288, *p* < 0.05) and premenopausal status (*β* = 0.300, *p* < 0.05) in adjusted models. A group composed of dairy products and legumes was correlated with the overexpression of *RhoA* (*β* = 0.249, *p* < 0.05) and *VEGF-A* (*β* = 0.297, *p* < 0.05) in VI+.

**Conclusions:**

Based on the multivariate findings, the dietary protein could associate with the overexpression of *RhoA* and *VEGF-VEGFR2* in favor of lymphatic and vascular metastasis in BC patients.

**Electronic supplementary material:**

The online version of this article (10.1186/s12263-019-0645-7) contains supplementary material, which is available to authorized users.

## Introduction

Breast cancer (BC) is the most frequently diagnosed malignancy in females in many countries [1]. In the recent decade, the prevalence rate of BC has been increasing rapidly among Iranian women [2]. Even lately, BC is the main leading cause of female cancer-related mortality [2]. Metastasis is a critical event in malignancy addressed as an indicator of poor prognosis and causes the vast majority of cancer-related deaths [3].

Sufficient epidemiologic evidence has revealed the association between some lifestyle-related risk factors and the BC risk [4]. However, it is far less understood how dietary factors can take part in cancer progression toward the formation of metastasis [5]. It is widely studied that breast carcinogenesis seems likely attributable to the high dietary intake of total protein, especially from animal sources [5–7]. Lately, in a meta-analysis of prospective studies, it has been documented that higher protein intake from red and processed meat is a potential risk factor for BC [6]. Moreover, in a large prospective cohort study, Cho et al. [7] found out that red meat intake strongly can elevate BC risk in pre-menopause women who had tumors with hormone receptor-positive status. In Nurses’ Health Study II during 20 years follow-up, it has been reported that high red meat intake in early adulthood might increase the risk of BC in later life [8]. Moreover, despite the previous findings suggested an association between protein intake and increased risk of metastasis [9, 10], a few experimental studies showed the effects of high protein content in association with augmented molecular alterations in promoting metastasis [11].

A review has highlighted that signaling pathways leading to cytoskeletal reprogramming are vital for cancerous cellular motility [12]. Rho (Ras homologous) is a superfamily of small GTPase, involved *Ras homologous gene family member A* (*RhoA*) as a key element function in neoplastic invasion and controlling cellular morphogenesis [13, 14]. Many in vitro experimental studies provide insights into the active contribution of upregulated *RhoA* in neoplastic propagation through holding the rearrangement of cytoskeletons, cellular motility, and subsequently cancer invasion [13]. On the one hand, Rho proteins modulate both F-actin formation and myosin activation, through *RhoA*-*Rho kinase* (*ROCK*) signaling pathway turned out as a molecular switch to catalyze GTP-GDP exchange [15]. The configuration of active GTP bound allows Rho protein to regulate signal transduction [15]. The phosphatidylinositol-3 kinase (PI3K) activates the *RhoA-ROCK* signaling pathway [12]. On the other hand, *hypoxia-inducible factor 1* (*HIF-1*)-dependent *RhoA-ROCK* signaling might result in enhanced BC cellular motility thereby increasing the risk of invasion and metastasis [14]. It is well-established that *RhoA* overexpressed in breast tumors [16]. However, a few clinical trials indicated that dietary protein intake can affect *RhoA* expression. Hebels et al. [17] showed that dietary intervention by red meat (7 days) in patients with irritable bowel disease can increase the expression levels of *RhoA* in colon tissue. High intake of animal proteins could increase the acid load of the blood, whereby low pH might be a stimulus for the formation of stress fiber mediated by *RhoA* [18]. To our knowledge, epidemiologic study to support the association of protein intake (the type of protein) and the transcription status of *RhoA* in breast tumors does not exist.

Angiogenesis is the formation of new blood vessels from the former vasculature, which is crucial in the propagation of tumoral cells to be enabled to grow in other anatomic sites and forming distant metastasis [19, 20]. Silent angiogenesis is precisely regulated by pro- and anti-angiogenic factors in healthy tissue, whereas pathologic angiogenesis potentiates tumorigenesis linked to the distorted balance of angiogenic variables grow endothelial cells [21]. *Vascular endothelial growth factor* (*VEGF*) is essential for growth and survival of endothelial cells accounted for promoting pathological angiogenesis [21, 22]. The family *VEGF* proteins are the major regulators of lymphatic and blood vessel formations [20]. While *VEGF-C* and *VEGF-D* considered being involved most often in lymphangiogenesis, *VEGF-A* is the predominant member of the *VEGF* family that regulates vasculogenesis essential in both physiologic and pathological angiogenesis [20]. *VEGF-A* could also induce lymphangiogenesis [23]. *VEGF receptor-2* (*VEGFR2*), a tyrosine kinase receptor, is distributed in all endothelial cells as well as cells of lymphatics capillaries and mediates the signaling pathway activated by *VEGF-A* as a ligand [23, 24]. *VEGF-A* is secreted by normal and malignant cells, and its overexpression was reported evidently in breast tumors [22]. Overregulated *VEGF-A* is significantly associated with poor prognosis in BC [22]. Hypoxia plays a major role in the overexpression of *VEGF* in breast tumors, because of the binding site on the promoter of *VEGF* gene for *HIF-1* [25]. In the absence of *HIF-1*, PI3K/*Rho*/*ROCK*/c-MYC pathway overrides the effect of *VEGF* by regulating a *cis*-regulatory element located in the *VEGF* gene promoter [12]. Much evidence suggested that high animal protein intake can raise the circulating levels of insulin-like growth factor-1 (IGF-1) that plays an important role in breast tumor progression [10, 26]. IGF-1-dependent PI3K/AKT/mammalian target of rapamycin (mTOR) signaling is a critical transcriptional activator pathway conceivably accounted for *VEGF* upregulation [10, 26]. No published data indicated whether the dietary protein can associate with the alterations in transcription of *VEGF-A* and *VEGFR2* as a nutrigenomic model may interact in lymphangiogenesis.

Given the fact that overexpression of *RhoA* and *VEGF-VEGFR2* is contributed to poor prognosis in malignancies [13, 22, 27] and evidence for linking protein intake and BC risk is often rare in epidemiologic studies [5], there is a need to investigate the nutrigenomic aspect of angiogenesis in a population of BC patients. Therefore, we aimed to study the association between dietary sources of protein and *RhoA*, *VEGF-A*, and *VEGFR2* expression levels in primary BC patients.

## Materials and methods

### Study population

We conducted a consecutive case series study, and 177 eligible newly diagnosed BC patients within the age range of 19–73 years old were recruited from May 2011 to November 2016 at Noor-Nejat Private Hospital in Tabriz, northwest of Iran. The inclusion criteria were histopathological confirmation of primary BC, no chronic inflammation, not vegetarian, not pregnant or a breastfed mother, and no longitudinal usage of drugs (e.g., methotrexate, sulfasalazine, anticonvulsants, and contraceptive drugs). The exclusion criteria consisted of a history of confirmed malignancy during life, history of adjuvant therapy, and history of breast benign disease. Also, those who had a body mass index above 45 kg/m^2^ were excluded from the study. Individuals’ pathological data were recorded from their medical history including histological tumor grade, tumor size, and histopathological subtype (ductal and non-ductal) and immunohistochemistry data such as estrogen receptor (ER), progesterone receptor (PR), and human epidermal growth factor receptor-2 (HER2-neu).

### Dietary assessment

Face-to-face interview with each participant was conducted by well-trained interviewers. Food frequency questionnaire (FFQ) with 136 food items was used to assess the dietary intake of protein. The accuracy of FFQ-based dietary intake measures of cobalamin and its association with a dietary group of protein had been met in previous studies [28–30]. Food intake of participants was questioned on a timely basis of daily, weekly, monthly, and yearly. FFQ was completed for the previous year. The amount of average daily intake of a nutrient was estimated by multiplying the time-dependent frequency of food intake to the amount of consumption in grams. Portion size was defined based on the common household utensil and then converted to gram using a standard reference value [31]. A set of photographs showing the major food groups was on hand to express better the consumption magnitude for the food items. Nutritionist IV software (ver.3.5.2; 1994, N-squared computing, San Bruno, USA) was used to compute the intake levels of total calories, macronutrients, and fiber. Total calorie intake was adjusted in terms of nutrient density as described by Willett [32].

### Extraction of mRNA and quantitative real-time reverse transcriptase polymerase chain reaction

Fresh tissues (tumor and adjacent normal tissues) were collected after dissection and carried in liquid nitrogen to store at − 70 °C. Based on microscopic examination, tumor cells on average constituted > 85% tissue sections. Total mRNA was extracted from malignant tissues by means of QIAzol (Qiagen, USA), and RNA cleanup was carried out using the RNeasy Mini Kit (Qiagen, USA). The quantity of mRNA was measured using NanoDrop 2000 (Thermo Scientific, Germany). For complementary DNA (cDNA) synthesis, samples were synchronized at the desired concentration and subsequently reverse-transcribed by QuantiTect (Qiagen, USA) reverse transcriptase with the integral removal of genomic DNA contamination. The threshold cycle (Ct) and subsequently relative expression levels were measured using qRT-PCR by means of LightCycler 480II (Roche, Germany). Primer sets were designed for human *RhoA* (F: 5′AAGCAGGTAGAGTTGGCTTTGTG3′; R: 5′-ATCGGTATCTGGGTAGGAGAGG-3′), *VEGF-A* (F: 5′-CTACCTCCACCATGCCAAGT-3′; R: 5′-CCACTTCGTGATGATTCTGC-3′), and *VEGFR2* (F: 5′-CATAGTTGTCGTTGTAGGGTA-3′; R: 5′-CATTTAGTTCAGTTCTTGCT-3′). The reaction master mix contained cDNA (~ 200 ng/μL), 10 × SYBER Green (Nanohelix, South Korea), and primers (~ 200–600 pg/μL) were used to have a reaction in a total volume of 25 μL. Thermal cycling included a first denaturation step at 95 °C for 15 min, followed by 40 cycles consisting of 24 s at 95 °C and 35 s at 62 °C. Duplicate reactions were carried out for a single sample. Quantification of fold changes was computed using 2^−ΔΔct^ formula. The expression of the *hypoxanthine-guanine phosphoribosyltransferase gene* (*HGPRT*; F: 5′-TGGACAGGACTGAACGTCTT-3′; R: 5′-CCAGCAGGTCAGCAAAGAAT-3′) was used as an internal normalizing control.

### Statistical analysis

Sample size was calculated based on the formula of mean comparisons and reach 100 subjects after considering the level of significance of *α* = 0.05 (two-sided), statistical power (1 − *β*) = 80%, and comparing the mean (SD) relative expression levels of intercellular adhesion molecule-1 (*ICAM1*) gene (high fiber 1.77 ± 0.47 vs. low fiber 1.94 ± 0.38) provided by Hermsdorff et al. [33]. On the other hand, for conducting principal component factor analysis (PCA), Gorsuch [34] and Hair et al. [35] recommended that sample size can be considered at least 100. A ratio of a sample size to observe variables was also highly suggested to be considered at 10 [35, 36]. Taken together, to meet the needed sample size for conducting PCA and to generalize the findings from a sample to a wider primary population, the sample size was estimated to be 177 subjects with BC by considering the necessity of keeping potential covariates controlled in the analysis. The normal distribution of quantitative variables was assessed by using the Kolmogorov-Smirnov test and plotting histogram. Outlier data were assigned by the plotting box plot and removed. Linear regression analysis was carried out to evaluate the correlation magnitude between protein intakes from different dietary sources and fold changes of studied genes. Standardized *β* coefficients (*β*) and adjusted *β* (*β*_adj._) were obtained from crude (unadjusted) and multivariate (adjusted) regression models, respectively. Models detailing the multivariate linear regression analyses consisted of following variables, just in case, when basic covariates were inter-correlated. The covariates were listed as follow: daily intake of energy (kcal/day); fat (g/day); saturated fat (g/day); cholesterol (mg/day); iron (mg/day); folate (μg/day); dietary, crude, soluble, and insoluble fiber (g/day); weight (kg); waist circumference (cm); hip circumference (cm); waist to hip ratio; body mass index (BMI; kg/m^2^); tumor size (cm); age at diagnosis (years); age at menarche (years); age at first childbirth (years); number of abortion; number of pregnancies; mean duration of breastfeeding (months); and mean duration of oral contraceptive usage (months). The PCA was carried out to derive appropriate dietary protein components (patterns). Accordingly, eight groups from different dietary protein sources were included in PCA. The eigenvalue is a parameter estimated to represent the sum of the variance of all the variables that can be explained by a given principal component and must be greater than 1.00, and the scree plot was applied to simplify making a decision in determining the number of components to be retained [35]. Factor loadings, the correlation between each variable and a certain component, are presented in the component matrix [35]. Since rotation is an important procedure to interpret the retained component, an orthogonal rotation in terms of the varimax procedure with Kaiser’s normalization was carried out [35]. Based on the rotated component matrix, a factor score for each subject on each component is computed to calculate individual’s scores on each variable involved in a component [35]. Indeed, an individual’s score obtained on each variable included in a component was multiplied by the factor loading for the particular variable. The sum of a person’s factor score on a component was then calculated. Subsequently, linear regression analysis was carried out to evaluate the correlation magnitude between the factor scores estimated for each component (pattern of protein sources) and fold changes of studied genes. Scatter plot was used to illustrate the correlations between the dietary patterns of protein and studied genes. Logistic regression analysis was performed to measure the odds ratio (OR) and 95% confidence interval (95% CI). Subgroup analyses based on the dichotomous status of the hormonal receptor of breast tumors (ER, PR, and HER-2) were done to control possibly the related potential effects. Further stratification analyses were relevant to the status of clinical outcomes such as tumor grade, vascular invasion (VI), and involvement of axillary lymph node metastasis (ALNM) to show whether an interested nutrigenomic correlation could be assigned in certain clinicopathological features of BC disease. Data analyses were carried out with the SPSS statistical software, version 16.0 (SPSS Inc., Chicago, IL, USA). All *p* values were two-tailed, and below 0.05 was considered statistically significant.

## Results

General demographic and descriptive characteristics of 177 study participants are shown in Table 1. The mean age at diagnosis of this study population was 46.67 ± 9.03 years. The relative number of premenopausal participants was 65.5% (116/177). The relative frequency of hormone receptor-positive tumors included 86.2% (131/152) of ER+, 84.8% (128/151) of PR+, and 18.1% (27/149) of HER2+ in the whole study population (*p* < 0.001). Histopathologic outcomes showed 81% (94/116) of patients with VI+ and 64.3% (99/154) with involvement of the ALNM in all available recorded data. Tumor grade II was significantly constituted in 73.3% (107/146) of study subjects.

**Table 1:** General characteristics of breast cancer patients in the study (*N* = 177)

Characteristics	Total patients	Relative frequency	*p* value
Age at diagnosis			
Mean ± SD	46.67 ± 9.03		
< 46	99	56.3	
≥ 46	77	43.8	0.097
Menopausal status			
Premenopausal	116	65.5	
Postmenopausal	61	34.5	< 0.001
Estrogen receptor			
Positive	131	86.2	
Negative	21	13.8	< 0.001
Progesterone receptor			
Positive	128	84.8	
Negative	23	15.2	< 0.001
Human epidermal growth factor receptor 2			
Positive	27	18.1	
Negative	122	81.9	< 0.001
Vascular invasion			
Positive	94	81	
Negative	22	19	< 0.001
Axillary lymph node metastasis			
Positive	99	64.3	
Negative	55	35.7	< 0.001
Grade			
I	24	16.4	
II	107	73.3	
III	15	10.3	< 0.001
BMI at diagnosis			
Normal	26	16	
Overweight	79	48.5	
Obese	58	35.6	< 0.001
Oral contraceptive use			
Yes	40	22.7	
No	136	77.3	< 0.001

Some missing data exist in general characteristics and histopathological status

*Chi-square test was performed to compare the proportion of values between the categories

### The correlation of protein intakes and fold changes of *RhoA*, *VEGF-A*, and *VEGFR2*

Findings of univariate and multivariate linear regression analyses to show the association between participant’s protein intake and fold changes in the expression of *RhoA*, *VEGF-A*, and *VEGFR2* were summarized in Table 2. Moreover, findings of linear regression analyses to show the correlation of dietary proteins with fold changes of genes of interest were presented based on hormonal receptor status (Tables 3 and 4) and clinical-pathological relevant outcomes (Tables 5 and 6).

**Table 2:** Correlations between intake levels of protein from different sources and fold change expression of *RhoA*, *VEGF-A*, and *VEGFR2* in the study population

Dietary variables	Fold change of *RhoA* (*n* = 167)				Fold change of *VEGF− A* (*n* = 169)				Fold change of *VEGFR2* (*n* = 160)			
	*β* ^§^	*p* value	*β*_adj._^¥^	*p* value	*β*	*p* value	*β*_adj._	*p* value	*β*	*p* value	*β*_adj._	*p* value
Total protein*	0.112	0.160	*0.178* ^a^	*0.044*	0.014	0.854	*0.200* ^n^	*0.029*	0.094	0.231	*0.217* ^aa^	*0.013*
Animal protein*	0.106	0.203	*0.192* ^b^	*0.043*	0.078	0.328	0.100^o^	0.246	*0.199*	*0.012*	*0.237* ^bb^	*0.007*
Plant protein*	− 0.049	0.547	− 0.139^c^	0.145	0.043	0.581	0.025^p^	0.769	− 0.038	0.639	− 0.048^cc^	0.567
Red meat	0.151	0.053	*0.348* ^d^	*0.001*	0.135	0.081	*0. 262* ^q^	*0.001*	*0.210*	*0.006*	*0.304* ^dd^	*< 0.001*
Processed red meat	0.045	0.569	0.086^e^	0.326	0.082	0.283	*0.184* ^r^	*0.043*	0.113	0.147	*0.191* ^ee^	*0.039*
Poultries	− 0.107	0.192	− 0.148^f^	0.106	− 0.016	0.833	− 0.086^s^	0.331	− 0.008	0.919	− 0.053^ff^	0.545
Seafood	− 0.053	0.499	− 0.080^g^	0.368	0.133	0.105	*0.180* ^t^	*0.038*	0.060	0.441	0.119^gg^	0.185
Dairy products	0.082	0.376	0.175^h^	0.133	0.131	0.085	*0.183* ^u^	*0.030*	0.049	0.539	0.101^hh^	0.283
Legumes	0.026	0.743	0.009^i^	0.918	− 0.097	0.214	− 0.127^v^	0.120	− 0.089	0.267	− 0.159^ii^	0.082
Nuts and cereals	− 0.129	0.100	*− 0.180* ^j^	*0.034*	0.088	0.224	− 0.015^w^	0.864	− 0.103	0.186	− 0.150^jj^	0.073
Residual protein	0.107	0.182	0.168^k^	0.053	0.117	0.165	*0.206* ^x^	*0.029*	0.001	0.989	0.110^kk^	0.248
Total protein/dietary fiber	0.131	0.097	*0.216* ^l^	*0.014*	− 0.115	0.138	0.005^y^	0.953	0.077	0.337	0.171^ll^	0.057
Animal protein/plant protein	0.069	0.399	*0.227* ^m^	*0.010*	0.001	0.981	0.100^z^	0.261	*0.213*	*0.007*	*0.283* ^mm^	*0.001*

*Energy-adjusted variables in terms of nutrient density was estimated. ^§^Values are expressed as *β* from a simple linear regression model. ^¥^Values are expressed as *β* from multivariate linear regression-adjusted model. Dietary variables adjusted for the following: ^a^Mean duration of breastfeeding (months), BMI (kg/m^2^), and crude fiber (g/day). ^b^Fat intake (g/day), crude fiber (g/day), the age of first childbirth (years), tumor size (cm), age at diagnosis (years), and the number of pregnancies. ^c^Energy intake (kcal/day), dietary fiber (g/day), and waist to hip ratio. ^d^Energy intake (kcal/day), the mean duration of breastfeeding (months), tumor size (cm), and BMI (kg/m^2^). ^e^Energy intake (kcal/day) and tumor size (cm). ^f^Log transformed and adjusted for fat intake (g/day), the age of menarche (years), and tumor size (cm). ^g^Age of first childbirth (years), the age of menarche (years), and waist circumference (cm). ^h^Log transformed and adjusted for energy intake (kcal/day), BMI (kg/m^2^), tumor size (cm), and age of menarche (years). ^i^BMI (kg/m^2^), tumor size (cm), and carbohydrate intake (g/day). ^j^Log transformed and adjusted for fat intake (g/day) and mean duration of breastfeeding (months). ^k^Mean duration of breastfeeding (months), physical activity rate and OCP usage. ^l^Carbohydrate intake (g/day), the age of menarche (years), and mean duration of breastfeeding (months). ^m^Fat intake (g/day), the age of first childbirth (years), and the number of lactation (*n*). ^n^Fat intake (g/day), tumor size (cm), and carbohydrate intake (g/day). ^o^Fat intake (g/day) and tumor size (cm). ^p^Dietary fiber intake (g/day) and waist to hip ratio. ^q^Fat intake (g/day). ^r^Fat intake (g/day), red meat intake (g/day), the age of first childbirth (years), waist to hip ratio, and age of menarche (years). ^s^Fat intake (g/day), mean duration of breastfeeding (months), and BMI (kg/m^2^). ^t^Log transformed and adjusted for energy intake (kcal/day) and mean duration of breastfeeding (months). ^u^Fat intake (g/day), red meat intake (g/day), number of pregnancies (*n*), and physical activity rate. ^v^Log transformed and adjusted for energy intake (kcal/day), insoluble fiber intake (g/day), and age at diagnosis (years). ^w^Energy intake (kcal/day), insoluble fiber (g/day), and waist to hip ratio. ^x^Fat intake (g/day), BMI (kg/m^2^), and tumor size (cm). ^y^Fat intake (g/day) and tumor size (cm). ^z^Fat intake (g/day), the age of first childbirth (years), and waist to hip ratio. ^aa^Dietary fiber intake (g/day), fat intake (g/day), and tumor size (cm). ^bb^Mean duration of breastfeeding (months) and tumor size (cm). ^cc^Fat intake (g/day) and age of menarche (years). ^dd^Fat intake (g/day), BMI (kg/m^2^), and tumor size (cm). ^ee^Red meat intake (g/day), fat intake (g/day), and tumor size (cm). ^ff^Fat intake (g/day), soluble fiber (g/day), and waist to hip ratio. ^gg^Energy intake (kcal), mean duration of breastfeeding (months), and tumor size (cm). ^hh^Red meat intake (g/day), mean duration of breastfeeding (months), physical activity rate, and tumor size (cm). ^ii^Log transformed and adjusted for fat intake (g/day), waist to hip ratio, and tumor size (cm). ^jj^Log transformed and adjusted for waist circumference (cm). ^kk^Fat intake (g/day), tumor size (cm), and waist circumference (cm). ^ll^Carbohydrate intake (g/day), the age of menarche (years), and waist to hip ratio. ^mm^Fat intake (g/day) and BMI (kg/m^2^)

**Table 3:** Correlations between intake levels of protein from different sources and fold change expression of *RhoA*, *VEGF-A*, and *VEGFR2* within each category of ER status

Dietary variables	Fold change of *RhoA*								Fold change of *VEGF-A*								Fold change of *VEGFR2*							
	ER+ (*n* = 123)				ER− (*n* = 20)				ER+ (*n* = 118)				ER− (*n* = 18)				ER+ (*n* = 115)				ER− *(n* = 14)			
	*β* ^**§**^	*p* value	*β*_adj._^**¥**^	*p* value	*β*	*p* value	*β*_adj._	*p* value	*β*	*p* value	*β*_adj._	*p* value	*β*	*p* value	*β*_adj._	*p* value	*β*	*p* value	*β*_adj._	*p* value	*β*	*p* value	*β*_adj._	*p* value
Total protein*	0.044	0.642	0.119^a^	0.260	0.111	0.651	0.007	0.977	*0.197*	*0.032*	0.207^n^	0.102	− 0.063	0.834	− 0.046	0.919	0.075	0.416	0.111^aa^	0.271	− 0.264	0.343	− 0.293	0.471
Animal protein*	0.130	0.181	*0.230* ^b^	*0.045*	− 0.07	0.755	*0.588*	*0.009*	0.119	0.199	0.098^o^	0.325	0.385	0.127	0.149	0.663	*0.253*	*0.005*	*0.299* ^bb^	*0.003*	− 0.263	0.344	− 0.444	0.357
Plant protein*	− 0.041	0.663	− 0.111^c^	0.292	− 0.018	0.941	− 0.247	0.419	0.144	0.132	0.137^p^	0.162	− 0.294	0.237	− 0.241	0.354	0.045	0.629	− 0.005^cc^	0.961	− 0.164	0.560	− 0.110	0.744
Red meat	*0.245*	*0.007*	*0.297* ^d^	*0.002*	− 0.151	0.538	− 0.091	0.717	*0.274*	*0.002*	*0.283* ^q^	*0.002*	0.121	0.643	0.318	0.289	*0.269*	*0.002*	*0.315* ^dd^	*0.001*	− 0.121	0.656	− 0.050	0.903
Processed red meat	0.056	0.546	0.087^e^	0.388	− 0.342	0.151	− 0.447	0.169	0.102	0.250	0.116^r^	0.294	0.124	0.624	0.013	0.973	0.068	0.452	0.133^ee^	0.186	*0.621*	*0.010*	0.643	0.126
Poultries	− 0.114	0.231	− 0.177^f^	0.088	− 0.019	0.940	*0.866*	*0.009*	− 0.070	0.441	− 0.071^s^	0.489	− 0.150	0.553	− 0.043	0.901	− 0.023	0.806	− 0.041^ff^	0.692	− 0.226	0.401	− 0.531	0.150
Seafood	− 0.049	0.597	− 0.072^g^	0.496	− 0.221	0.350	− 0.406	0.194	0.076	0.425	0.138^t^	0.188	− 0.350	0.183	− 0.351	0.247	0.051	0.572	0.095^gg^	0.364	− 0.135	0.618	0.059	0.827
Dairy products	0.076	0.426	0.153^h^	0.172	0.280	0.232	0.673	0.076	0.109	0.219	0.126^u^	0.187	*0.486*	*0.035*	*0.629*	*0.050*	− 0.058	0.530	− 0.039^hh^	0.711	0.467	0.068	0.646	0.193
Legumes	0.023	0.799	0.003^i^	0.976	0.408	0.074	0.673	0.124	− 0.064	0.479	− 0.081^v^	0.408	0.118	0.630	− 0.145	0.709	− 0.150	0.102	− 0.177^ii^	0.067	0.398	0.126	0.697	0.051
Nuts and cereals	− 0.074	0.419	*− 0.227* ^j^	*0.045*	− 0.249	0.289	− 0.231	0.359	0.133	0.131	0.116^w^	0.283	− 0.257	0.288	− 0.387	0.099	− 0.112	0.212	− 0.130^jj^	0.166	− 0.053	0.844	− 0.307	0.248
Residual protein	0.06	0.527	0.135^k^	0.229	0.01	0.967	0.329	0.316	0.169	0.082	*0.311* ^x^	*0.004*	− 0.150	0.578	− 0.100	0.786	− 0.034	0.724	0.090^kk^	0.405	0.068	0.816	− 0.330	0.101
Total protein/dietary fiber	0.146	0.117	*0.249* ^l^	*0.020*	0.402	0.088	0.382	0.202	0.034	0.714	0.020^y^	0.844	− 0.195	0.438	− 0.137	0.689	0.108	0.243	0.197^ll^	0.056	− 0.130	0.658	− 0.105	0.779
Animal protein/plant protein	0.044	0.650	0.160^m^	0.133	0.081	0.741	0.132	0.708	− 0.012	0.897	0.076^z^	0.471	0.292	0.242	0.344	0.205	*0.253*	*0.006*	*0.331* ^mm^	*< 0.001*	− 0.251	0.348	− 0.254	0.406

*ER* estrogen receptor

*Energy-adjusted variables in terms of nutrient density were estimated. ^§^Values are expressed as *β* from a simple linear regression model. ^¥^Values are expressed as *β* from multivariate linear regression adjusted model. Dietary variables adjusted for the following: ^a^Mean duration of breastfeeding (months), BMI (kg/m^2^), and crude fiber (g/day). ^b^Fat intake (g/day), crude fiber (g/day), BMI (kg/m^2^), age first childbirth (years), and number of pregnancies. ^c^Energy intake (kcal/day), dietary fiber, and BMI (kg/m^2^). ^d^Energy intake (kcal/day). ^e^Energy intake (kcal/day) and tumor size (cm). ^f^Log transformed and adjusted for fat intake (g/day), the age of menarche (years), and tumor size (cm). ^g^Age of first childbirth (years), the age of menarche (years), and waist circumference (cm). ^h^Log transformed and adjusted for energy intake (kcal/day), BMI (kg/m^2^), tumor size (cm), and age of menarche (years). ^i^BMI (kg/m^2^), tumor size (cm), and carbohydrate intake (g/day). ^j^Log transformed and adjusted for fat intake (g/day), the mean duration of breastfeeding (months), BMI (kg/m^2^), and tumor size (cm). ^k^Mean duration of breastfeeding (months), BMI (kg/m^2^), fat intake (g/day), and soluble fiber (g/day). ^l^Carbohydrate intake (g/day), the age of menarche (years), and mean duration of breastfeeding (months). ^m^Fat intake (g/day), the age of first childbirth (years), and number of lactation (*n*). ^n^Fat intake (g/day), tumor size (cm), and carbohydrate intake (g/day). ^o^Fat intake (g/day) and tumor size (cm). ^p^Dietary fiber intake (g/day) and waist to hip ratio. ^q^Fat intake (g/day). ^r^Fat intake (g/day), red meat intake (g/day), the age of first childbirth (years), waist to hip ratio, and age of menarche (years). ^s^Fat intake (g/day), mean duration of breastfeeding (months), and BMI (kg/m^2^). ^t^Log transformed and adjusted for energy intake (kcal/day) and mean duration of breastfeeding (months). ^u^Fat intake (g/day), red meat intake (g/day), number of pregnancies (*n*), and physical activity rate. ^v^Log transformed and adjusted for energy intake (kcal/day), insoluble fiber intake (g/day), and age at diagnosis (years). ^w^Energy intake (kcal/day), insoluble fiber (g/day), and waist to hip ratio. ^x^Fat intake (g/day), BMI (kg/m^2^), and tumor size (cm). ^y^Fat intake (g/day) and tumor size (cm). ^z^Fat intake (g/day), the age of first childbirth (years), and waist to hip ratio. ^aa^Dietary fiber intake (g/day), fat intake (g/day), and tumor size (cm). ^bb^Mean duration of breastfeeding (months) and tumor size (cm). ^cc^Fat intake (g/day) and age of menarche (years). ^dd^Fat intake (g/day), BMI (kg/m^2^), and tumor size (cm). ^ee^Red meat intake (g/day), fat intake (g/day), and tumor size (cm). ^ff^Fat intake (g/day), soluble fiber (g/day), and waist to hip ratio. ^gg^Energy intake (kcal), mean duration of breastfeeding (months), and tumor size (cm). ^hh^Red meat intake (g/day), mean duration of breastfeeding (months), physical activity rate, and tumor size (cm). ^ii^Log transformed and adjusted for fat intake (g/day), waist to hip ratio, and tumor size (cm). ^jj^Log transformed and adjusted waist circumference (cm). ^kk^Fat intake (g/day), tumor size (cm), and waist circumference (cm). ^ll^Carbohydrate intake (g/day), the age of menarche (years), and waist to hip ratio. ^mm^Fat intake (g/day) and BMI (kg/m^2^)

**Table 4:** Correlations between intake levels of protein from different sources and fold change expression of *RhoA*, *VEGF-A*, and *VEGFR2* within each category of PR status

Dietary variables	Fold change of *RhoA*								Fold change of *VEGF-A*								Fold change of *VEGFR2*							
	PR+ (*n* = 120)				PR− (*n* = 22)				PR+ (*n* = 115)				PR− (*n* = 20)				PR+ (*n* = 113)				PR− (*n* = 15)			
	*β* ^§^	*p* value	*β*_adj._^¥^	*p* value	*β*	*p* value	*β*_adj._	*p* value	*β*	*p* value	*β*_adj._	*p* value	*β*	*p* value	*β*_adj._	*p* value	*β*	*p* value	*β*_adj._	*p* value	*β*	*p* value	*β*_adj._	*p* value
Total protein*	0.047	0.622	0.116^a^	0.270	− 0.104	0.628	0.130	0.579	*0.211*	*0.023*	*0.296* ^n^	*0.005*	− 0.064	0.782	− 0.092	0.802	0.076	0.410	0.113^aa^	0.266	− 0.262	0.309	− 0.318	0.376
Animal protein*	0.126	0.200	0.160^b^	0.145	0.342	0.102	*0.594*	*0.003*	0.127	0.177	0.105^**o**^	0.296	0.317	0.174	0.257	0.374	*0.250*	*0.006*	*0.296* ^bb^	*0.004*	− 0.213	0.413	− 0.390	0.189
Plant protein*	− 0.048	0.614	− 0.118^c^	0.286	− 0.048	0.823	− 0.222	0.443	0.169	0.066	0.173^p^	0.081	− 0.306	0.177	− 0.273	0.264	0.039	0.678	− 0.008^cc^	0.940	− 0.109	0.676	− 0.060	0.838
Red meat	*0.248*	*0.007*	*0.305* ^d^	*0.002*	− 0.126	0.586	− 0.109	0.635	*0.259*	*0.004*	*0.271* ^q^	*0.004*	0.349	0.142	0.387	0.131	*0.267*	*0.003*	*0.315* ^dd^	*0.001*	− 0.113	0.665	− 0.049	0.897
Processed red meat	0.053	0.571	0.083^e^	0.413	− 0.303	0.182	− 0.346	0.227	0.109	0.226	0.133^r^	0.233	0.073	0.758	0.207	0.656	0.066	0.471	0.148^ee^	0.166	*0.639*	*0.006*	*0.918*	*0.044*
Poultries	− 0.115	0.234	− 0.180^f^	0.088	− 0.013	0.956	*0.702*	*0.019*	− 0.072	0.435	− 0.75^s^	0.466	− 0.075	0.753	0.143	0.614	− 0.026	0.782	− 0.040^ff^	0.699	− 0.229	0.376	− 0.522	0.132
Seafood	− 0.051	0.583	− 0.074^g^	0.490	− 0.167	0.459	− 0.381	0.185	0.077	0.425	0.138^t^	0.191	− 0.192	0.444	− 0.161	0.529	0.049	0.594	0.093^gg^	0.381	− 0.179	0.492	0.019	0.938
Dairy products	0.070	0.448	0.157^h^	0.162	0.298	0.179	0.373	0.101	0.118	0.189	0.145^u^	0.142	0.321	0.156	*0.615*	*0.024*	− 0.060	0.520	− 0.046^hh^	0.660	0.418	0.095	0.670	0.149
Legumes	0.020	0.827	0.000^i^	0.997	0.420	0.051	0.599	0.074	− 0.021	0.823	− 0.031^v^	0.755	− 0.063	0.787	− 0.336	0.330	− 0.148	0.112	− 0.174^ii^	0.074	0.314	0.220	*0.700*	*0.038*
Nuts and cereals	− 0.078	0.400	*− 0.225* ^j^	*0.049*	− 0.230	0.303	− 0.164	0.474	0.139	0.119	0.131^w^	0.230	− 0.211	0.358	− 0.334	0.175	− 0.116	0.202	− 0.129^jj^	0.171	0.019	0.941	− 0.254	0.271
Residual protein	0.063	0.509	0.137^k^	0.227	− 0.028	0.902	0.316	0.311	0.176	0.073	*0.336* ^x^	*0.002*	− 0.124	0.612	− 0.083	0.812	− 0.033	0.737	0.087^kk^	0.426	0.048	0.861	− 0.287	0.177
Total protein/dietary fiber	0.139	0.140	*0.229* ^l^	*0.034*	0.418	0.053	0.396	0.123	0.036	0.697	0.016^y^	0.876	− 0.142	0.538	− 0.184	0.535	0.097	0.300	0.172^ll^	0.095	− 0.115	0.672	− 0.467	0.233
Animal protein/plant protein	0.044	0.651	0.164^m^	0.129	0.074	0.750	0.087	0.765	− 0.018	0.847	0.063^z^	0.553	0.318	0.172	0.352	0.172	*0.252*	*0.006*	*0.331* ^mm^	*0.001*	− 0.245	0.343	− 0.217	0.452

*PR* progesterone receptor

*Energy-adjusted variables in terms of nutrient density was estimated. ^§^Values are expressed as *β* from a simple linear regression model. ^¥^Values are expressed as *β* from multivariate linear regression adjusted model. Dietary variables adjusted for the following: ^a^Mean duration of breastfeeding (months), BMI (kg/m^2^), and crude fiber (g/day). ^b^Fat intake (g/day), crude fiber (g/day), the age of first childbirth (years), BMI (kg/m^2^), the age first childbirth (years), and the number of pregnancies. ^c^Energy intake (kcal/day), dietary fiber, and BMI (kg/m^2^). ^d^Energy intake (kcal/day). ^e^Energy intake (kcal/day) and tumor size (cm). ^f^Log transformed and adjusted for fat intake (g/day), the age of menarche (years), and tumor size (cm). ^g^Age of first childbirth (years), the age of menarche (years), and waist circumference (cm). ^h^Log transformed and adjusted for energy intake (kcal/day), BMI (kg/m^2^), tumor size (cm), and age of menarche (years). ^i^BMI (kg/m^2^), tumor size (cm), and carbohydrate intake (g/day). ^j^Log transformed and adjusted for fat intake (g/day), the mean duration of breastfeeding (months), BMI (kg/m^2^), and tumor size (cm). ^k^Mean duration of breastfeeding (months), BMI (kg/m^2^), fat intake (g/day), and soluble fiber (g/day). ^l^Carbohydrate intake (g/day), the age of menarche (years), and mean duration of breastfeeding (months). ^m^Fat intake (g/day), the age of first childbirth (years), and the number of lactation (*n*). ^n^Fat intake (g/day), tumor size (cm), and carbohydrate intake (g/day). ^o^Fat intake (g/day) and tumor size (cm). ^p^Dietary fiber intake (g/day) and waist to hip ratio. ^q^Fat intake (g/day). ^r^Fat intake (g/day), red meat intake (g/day), the age of first childbirth (years), waist to hip ratio, and age of menarche (years). ^s^Fat intake (g/day), mean duration of breastfeeding (months), and BMI (kg/m^2^). ^t^Log transformed and adjusted for energy intake (kcal/day) and mean duration of breastfeeding (months). ^u^Fat intake (g/day), red meat intake (g/day), number of pregnancies (*n*), and physical activity rate. ^v^Log transformed and adjusted for energy intake (kcal/day), insoluble fiber intake (g/day), and age at diagnosis (years). ^w^Energy intake (kcal/day), insoluble fiber (g/day), and waist to hip ratio. ^x^Fat intake (g/day), BMI (kg/m^2^), and tumor size (cm). ^y^Fat intake (g/day) and tumor size (cm). ^z^Fat intake (g/day), the age of first childbirth (years), and waist to hip ratio. ^aa^Dietary fiber intake (g/day), fat intake (g/day), and tumor size (cm). ^bb^Mean duration of breastfeeding (months) and tumor size (cm). ^cc^Fat intake (g/day) and age of menarche (years). ^dd^Fat intake (g/day), BMI (kg/m^2^), and tumor size (cm). ^ee^Red meat intake (g/day), fat intake (g/day), and tumor size (cm). ^ff^Fat intake (g/day), soluble fiber (g/day), and waist to hip ratio. ^gg^Energy intake (kcal), mean duration of breastfeeding (months), and tumor size (cm). ^hh^Red meat intake (g/day), mean duration of breastfeeding (months), physical activity rate, and tumor size (cm). ^ii^Log transformed and adjusted for fat intake (g/day), waist to hip ratio, and tumor size (cm). ^jj^Log transformed and adjusted waist circumference (cm). ^kk^Fat intake (g/day), tumor size (cm), and waist circumference (cm). ^ll^Carbohydrate intake (g/day), the age of menarche (years), and waist to hip ratio. ^mm^Fat intake (g/day) and BMI (kg/m^2^)

**Table 5:** Correlations between intake levels of protein from different sources and fold change expression of *RhoA*, *VEGF-A*, and *VEGFR2* within each category of ALNM status

Dietary variables	Fold change of *RhoA*								Fold change of *VEGF-A*								Fold change of *VEGFR2*							
	ALNM+ (*n* = 92)				ALNM− (*n* = 53)				ALNM+ (*n* = 87)				ALNM− (*n* = 52)				ALNM+ (*n* = 87)				ALNM− (*n* = 46)			
	*β* ^**§**^	*p* value	*β*_adj._^**¥**^	*p* value	*β*	*p* value	*β*_adj._	*p* value	*β*	*p* value	*β*_adj._	*p* value	*β*	*p* value	*β*_adj._	*p* value	*β*	*p* value	*β*_adj._	*p* value	*β*	*p* value	*β*_adj._	*p* value
Total protein*	0.104	0.346	0.216^a^	0.079	− 0.03	0.819	− 0.177	0.291	0.013	0.908	0.088^n^	0.477	*0.321*	*0.017*	*0.339*	*0.019*	*0.257*	*0.015*	*0.295* ^aa^	*0.009*	0.095	0.503	0.050	0.747
Animal protein*	0.158	0.154	*0.238* ^b^	*0.041*	− 0.050	0.708	0.205	0.217	0.117	0.283	0.099^o^	0.382	0.117	0.400	0.107	0.461	− 0.060	0.580	− 0.059^bb^	0.593	*0.428*	*0.002*	*0.446*	*0.002*
Plant protein*	− 0.009	0.936	− 0.195^c^	0.168	0.062	0.635	− 0.181	0.301	0.090	0.408	0.091^p^	*0.425*	0.187	0.169	0.176	0.257	− 0.112	0.303	− 0.116^cc^	0.316	0.146	0.305	0.137	0.354
Red meat	0.182	0.085	*0.230* ^d^	*0.039*	− 0.027	0.841	0.073	0.632	*0.294*	*0.005*	*0.321* ^q^	*0.003*	0.197	0.143	0.221	0.115	− 0.078	0.460	− 0.013^dd^	0.909	*0.425*	*0.002*	*0.444*	*0.002*
Processed red meat	0.067	0.533	0.102^e^	0.374	− 0.005	0.973	0.006	0.967	0.058	0.577	0.047^r^	0.695	0.117	0.386	0.082	0.617	0.152	0.151	*0.265* ^ee^	*0.030*	0.066	0.641	0.202	0.186
Poultries	− 0.135	0.226	− 0.163^f^	0.185	− 0.089	0.543	− 0.139	0.372	− 0.018	0.862	− 0.039^s^	0.744	− 0.092	0.503	− 0.136	0.396	− 0.031	0.772	− 0.066^ff^	0.569	0.042	0.781	0.043	0.805
Seafood	− 0.034	0.747	− 0.083^g^	0.493	− 0.109	0.444	− 0.086	0.643	0.040	0.727	0.086^t^	0.496	0.124	0.396	0.141	0.380	− 0.055	0.600	− 0.013^gg^	0.911	0.140	0.322	0.101	0.520
Dairy products	0.132	0.215	*0.271* ^h^	*0.03*	− 0.035	0.806	0.024	0.889	0.157	0.132	0.203^u^	0.067	0.094	0.487	0.106	0.501	0.067	0.524	0.125^hh^	0.285	0.017	0.908	*0.458*	*0.003*
Legumes	0.021	0.841	0.005^i^	0.964	− 0.011	0.940	0.015	0.928	*0.237*	*0.025*	0.205^v^	0.095	− 0.196	0.145	− 0.267	0.147	− 0.154	0.155	− 0.201^ii^	0.089	− 0.007	0.959	− 0.095	0.560
Nuts and cereals	− 0.130	0.226	*− 0.239* ^j^	*0.043*	− 0.021	0.879	− 0.099	0.543	0.072	0.492	0.064^w^	0.574	0.080	0.547	− 0.019	0.912	0.020	0.193	0.091^jj^	0.413	− 0.169	0.227	− 0.219	0.153
Residual protein	0.128	0.250	0.228^k^	0.086	− 0.158	0.268	− 0.207	0.228	0.071	0.544	0.142^x^	0.249	0.258	0.080	0.272	0.107	0.077	0.493	0.184^kk^	0.121	− 0.058	0.709	0.123	0.491
Total protein/dietary fiber	0.177	0.102	*0.242* ^l^	*0.040*	− 0.033	0.802	0.119	0.488	0.006	0.954	− 0.055^y^	0.643	0.089	0.514	0.064	0.649	0.002	0.988	0.129^ll^	0.301	0.158	0.264	*0.303*	*0.041*
Animal protein/plant protein	0.173	0.111	*0.273* ^m^	*0.022*	− 0.029	0.853	0.002	0.993	− 0.017	0.871	− 0.032^z^	0.791	− 0.020	0.892	0.112	0.507	0.081	0.451	− 0.031^mm^	0.787	*0.300*	*0.038*	*0.388*	*0.011*

*ALNM* axillary lymph node metastasis

*Energy-adjusted variables in terms of nutrient density was estimated. ^§^Values are expressed as *β* from simple linear regression model. ^¥^Values are expressed as *β* from multivariate linear regression adjusted model. Dietary variables adjusted for the following: ^a^Mean duration of breastfeeding (months), BMI (kg/m^2^), OCP usage, and menopause status. ^b^Crude fiber (g/day) and age of first childbirth (years). ^c^Energy intake (kcal/day), dietary fiber (g/day), the age of menarche (years), menopause status, and BMI (kg/m^2^). ^d^Energy intake (kcal/day). ^e^Energy intake (kcal/day) and tumor size (cm). ^f^Log transformed and adjusted for fat intake (g/day), the age of menarche (years), and tumor size. ^g^Age of first childbirth (years), the age of menarche (years), and waist circumference (cm). ^h^Log transformed and adjusted for energy intake (kcal/day), BMI (kg/m^2^), the age of first childbirth (years), and mean duration of breastfeeding (months). ^i^BMI (kg/m^2^), tumor size (cm), and carbohydrate intake (g/day). ^j^Log transformed and adjusted for fat intake (g/day), the mean duration of breastfeeding (months), dietary fiber (g/day), and BMI (kg/m^2^). ^k^Mean duration of breastfeeding (months), BMI (kg/m^2^), fat intake(g/day), and soluble fiber (g/day). ^l^Carbohydrate intake (g/day), the age of menarche (years), and mean duration of breastfeeding (months). ^m^Fat intake (g/day), the age of first childbirth (years), and number of lactation (*n*). ^n^Fat intake (g/day), tumor size (cm), and carbohydrate intake (g/day). ^o^Fat intake (g/day) and tumor size (cm). ^p^Dietary fiber intake (g/day) and waist to hip ratio. ^q^Fat intake (g/day). ^r^Fat intake (g/day), red meat intake (g/day), the age of first childbirth (years), waist to hip ratio, and age of menarche (years). ^s^Fat intake (g/day), mean duration of breastfeeding (months), and BMI (kg/m^2^). ^t^Log transformed and adjusted for energy intake (kcal/day) and mean duration of breastfeeding (months). ^u^Fat intake (g/day), red meat intake (g/day), number of pregnancies (*n*), and physical activity rate. ^v^Log transformed and adjusted for energy intake (kcal/day), insoluble fiber intake (g/day), and age at diagnosis (years). ^w^Energy intake (kcal/day), insoluble fiber (g/day), and waist to hip ratio. ^x^Fat intake (g/day), BMI (kg/m^2^), and tumor size (cm). ^y^Fat intake (g/day) and tumor size (cm). ^z^Fat intake (g/day), the age of first childbirth (years), and waist to hip ratio. ^aa^Dietary fiber intake (g/day), fat intake (g/day), and tumor size (cm). ^bb^Mean duration of breastfeeding (months) and tumor size (cm). ^cc^Fat intake (g/day) and age of menarche (years). ^dd^Fat intake (g/day), BMI (kg/m^2^), and tumor size (cm). ^ee^Red meat intake (g/day), fat intake (g/day), and tumor size (cm). ^ff^Fat intake (g/day), soluble fiber (g/day), and waist to hip ratio. ^gg^Energy intake (kcal), mean duration of breastfeeding (months), and tumor size (cm). ^hh^Red meat intake (g/day), mean duration of breastfeeding (months), physical activity rate, and tumor size (cm). ^ii^Log transformed and adjusted for fat intake (g/day), waist to hip ratio, and tumor size (cm). ^jj^Log transformed and adjusted waist circumference (cm). ^kk^Fat intake (g/day), tumor size (cm), and waist circumference (cm). ^ll^Carbohydrate intake (g/day), the age of menarche (years), and waist to hip ratio. ^mm^Fat intake (g/day) and BMI (kg/m^2^)

**Table 6:** Correlations between intake levels of protein from different sources and fold change expression of *RhoA*, *VEGF-A*, and *VEGFR2* within each category of VI status

Dietary variables	Fold change of *RhoA*								Fold change of *VEGF-A*								Fold change of *VEGFR2*							
	VI+ (*n* = 89)				VI− (*n* = 21)				VI+ (*n* = 84)				VI− (*n* = 19)				VI+ (n = 83)				VI− (*n* = 18)			
	*β* ^**§**^	*p* value	*β*_adj._^**¥**^	*p* value	*β*	*p* value	*β*_adj._	*p* value	*β*	*p* value	*β*_adj._	*p* value	*β*	*p* value	*β*_adj._	*p* value	*β*	*p* value	*β*_adj._	*p* value	*β*	*p* value	*β*_adj._	*p* value
Total protein*	0.108	0.333	0.133^a^	0.261	− 0.227	0.336	0.113	0.701	0.088	0.425	0.161^n^	0.171	0.396	0.103	0.421	0.224	− 0.082	0.451	− 0.095^aa^	0.391	0.323	0.153	0.418	0.134
Animal protein*	*0.315*	*0.004*	*0.313* ^b^	*0.007*	− 0.038	0.883	0.121	0.646	0.141	0.197	0.119^o^	0.303	0.099	0.687	0.092	0.722	*0.441*	*< 0.001*	*0.444* ^bb^	*< 0.001*	− 0.022	0.925	0.042	0.881
Plant protein*	− 0.012	0.911	− 0.200^c^	0.155	− 0.181	0.446	− 0.182	0.550	0.026	0.812	− 0.012^p^	0.920	*0.490*	*0.024*	0.425	0.109	− 0.044	0.689	− 0.114^cc^	0.340	0.383	0.096	0.366	0.167
Red meat	*0.271*	*0.012*	*0.358* ^d^	*0.001*	0.002	0.994	− 0.026	0.913	*0.319*	*0.003*	*0.346* ^q^	*0.002*	0.339	0.122	0.331	0.148	*0.419*	*< 0.001*	*0.467* ^dd^	*< 0.001*	0.069	0.766	− 0.001	0.997
Processed red meat	0.004	0.973	0.042^e^	0.715	0.001	0.996	0.021	0.933	0.075	0.488	0.010^r^	0.944	0.381	0.082	0.223	0.506	− 0.020	0.852	− 0.005^ee^	0964	0.330	0.144	0.728	0.082
Poultries	− 0.104	0.360	− 0.131^f^	0.280	− 0.044	0.860	− 0.136	0.561	− 0.006	0.956	− 0.019^s^	0.874	− 0.096	0.696	− 0.355	0.400	0.137	0.204	0.069^ff^	0.554	− 0.151	0.550	− 0.013	0.966
Seafood	− 0.028	0.794	− 0.048^g^	0.698	− 0.070	0.770	− 0.132	0.692	0.073	0.618	0.100^t^	0.426	0.132	0.590	0.067	0.828	− 0.011	0.920	0.062^gg^	0.588	0.038	0.873	− 0.120	0.706
Dairy products	0.196	0.071	*0.275* ^h^	*0.035*	0.095	0.681	0.223	0.495	*0.225*	*0.032*	*0.263* ^u^	*0.020*	0.095	0.683	0.038	0.862	− 0.097	0.358	− 0.131^hh^	0.231	0.008	0.973	0.318	0.970
Legumes	0.036	0.743	0.019^i^	0.875	0.036	0.743	0.143	0.668	0.143	0.181	0.048^v^	0.712	0.036	0.882	− 0.072	0.840	− 0.099	0.354	− 0.002^ii^	0.985	− 0.233	0.337	− 0.582	0.083
Nuts and cereals	− 0.130	0.234	*− 0.234* ^j^	*0.045*	0.019	0.936	− 0.165	0.722	0.059	0.580	0.030^w^	0.800	− 0.032	0.887	− 0.038	0.902	− 0.065	0.541	− 0.076^jj^	0.485	− 0.130	0.573	− 0.173	0.519
Residual protein	0.090	0.418	0.163^k^	0.176	− 0.262	0.251	− 0.181	0.543	0.111	0.341	0.130^x^	0.270	0.314	0.219	0.413	0.252	− 0.170	0.140	− 0.026^kk^	0.841	0.090	0.714	0.110	0691
Total protein/dietary fiber	*0.215*	*0.050*	*0.305* ^l^	*0.010*	− 0.188	0.415	0.213	0.420	− 0.002	0.984	0.047^y^	0.693	*0.512*	*0.021*	*0.507*	*0.037*	− 0.084	0.452	0.056^ll^	0.654	0.365	0.113	0.405	0.149
Animal protein/plant protein	*0.246*	*0.026*	*0.290* ^m^	*0.018*	0.067	0.805	0.123	0.616	0.066	0.542	0.100^z^	0.409	− 0.118	0.620	0.019	0.953	*0.419*	*< 0.001*	*0.463* ^mm^	*< 0.001*	− 0.137	0.565	− 0.223	0.434

*VI* vascular invasion

*Energy-adjusted variables in terms of nutrient density was estimated. ^§^Values are expressed as *β* from a simple linear regression model. ^¥^Values are expressed as *β* from multivariate linear regression adjusted model. Dietary variables adjusted for the following: ^a^Mean duration of breastfeeding (months), BMI (kg/m^2^), and crude fiber (g/day). ^b^Fat intake (g/day), crude fiber (g/day), the age of first childbirth (years), tumor size (cm), the age at diagnosis (years), and number of pregnancies. ^c^Energy intake (kcal/day), dietary fiber(g/day), and waist/hip ratio. ^d^Energy intake (kcal/day). ^e^Energy intake (kcal/day) and tumor size (cm). ^f^Log transformed and adjusted for fat intake (g/day), the age of menarche (years), and tumor size (cm). ^g^Age of first childbirth (years), the age of menarche (years), and waist circumference (cm). ^h^Log transformed and adjusted for energy intake (kcal/day), BMI (kg/m^2^), tumor size (cm), and age of menarche (years). ^i^BMI (kg/m^2^), tumor size (cm), and carbohydrate intake (g/day). ^j^Log transformed and adjusted for fat intake (g/day) and mean duration of breastfeeding (months). ^k^Mean duration of breastfeeding (months), physical activity rate, and OCP usage. ^l^Carbohydrate intake (g/day), the age of menarche (years), and mean duration of breastfeeding (months). ^m^Fat intake (g/day), the age of first childbirth (years), and number of lactation (*n*). ^n^Fat intake (g/day), tumor size (cm)n and carbohydrate intake (g/day). ^o^Fat intake (g/day) and tumor size (cm). ^p^Dietary fiber intake (g/day) and waist to hip ratio. ^q^Fat intake (g/day). ^r^Fat intake (g/day), red meat intake (g/day), the age of first childbirth (years), waist to hip ratio, and age of menarche (years). ^s^Fat intake (g/day), mean duration of breastfeeding (months), and BMI (kg/m^2^). ^t^Log transformed and adjusted for energy intake (kcal/day) and mean duration of breastfeeding (months). ^u^Fat intake (g/day), red meat intake (g/day), number of pregnancies (*n*), and physical activity rate. ^v^Log transformed and adjusted for energy intake (kcal/day), insoluble fiber intake (g/day), and age at diagnosis (years). ^w^Energy intake (kcal/day), insoluble fiber (g/day), and waist to hip ratio. ^x^Fat intake (g/day), BMI (kg/m^2^), and tumor size (cm). ^y^Fat intake (g/day) and tumor size (cm). ^z^Fat intake (g/day), the age of first childbirth (years), and waist to hip ratio. ^aa^Dietary fiber intake (g/day), fat intake (g/day), and tumor size (cm). ^bb^Mean duration of breastfeeding (months) and tumor size (cm). ^cc^Fat intake (g/day) and age of menarche (years). ^dd^Fat intake (g/day), BMI (kg/m^2^), and tumor size (cm). ^ee^Red meat intake (g/day), fat intake (g/day), and tumor size (cm). ^ff^Fat intake (g/day), soluble fiber (g/day), and waist to hip ratio. ^gg^Energy intake (kcal), mean duration of breastfeeding (months), and tumor size (cm). ^hh^Red meat intake (g/day), mean duration of breastfeeding (months), physical activity rate, and tumor size (cm). ^ii^Log transformed and adjusted for fat intake (g/day), waist to hip ratio, and tumor size (cm). ^jj^Log transformed and adjusted waist circumference (cm). ^kk^Fat intake (g/day), tumor size (cm), and waist circumference (cm). ^ll^Carbohydrate intake (g/day), the age of menarche (years), and waist to hip ratio. ^mm^Fat intake (g/day) and BMI (kg/m^2^)

#### RhoA

High total amounts of protein intake (*β*_adj._ = 0.178, *p* = 0.044), animal proteins (*β*_adj._ = 0.192, *p* = 0.043), and red meat protein (*β*_adj._ = 0.348, *p* = 0.001) were observed to associate significantly with the overexpression of *RhoA*. The ratio of total protein to dietary fiber (TP:DF ratio, *β*_adj._ = 0.216, *p* = 0.014) and animal proteins to plant proteins (AP:PP ratio, *β*_adj._ = 0.227, *p* = 0.010) was positively associated with *RhoA* expression (Table 2). Moreover, protein intakes from nuts and cereals were inversely associated with *RhoA* expression in the whole sample population, when adjustments were made for confounders (*β*_adj._ = − 0.180, *p* = 0.034).

In case of ER+ feature, the multivariate-adjusted models showed that animal proteins (*β*_adj._ = 0.230, *p* = 0.045), red meat protein (*β*_adj._ = 0.297, 0.002), and the ratio of TP:DF (*β*_adj._ = 0.249, *p* = 0.020) could associate positively with *RhoA* expression (Table 3). In PR+ breast tumors, greater red meat consumed (*β*_adj._ = 0.305, *p* = 0.002) and high ratio of TP:DF (*β*_adj._ = 0.229, *p* = 0.034) were associated with higher fold changes of *RhoA* expression (Table 4). Protein obtained from nuts and cereals correlated inversely with fold changes of *RhoA* expression (ER+: *β*_adj._ = − 0.227, *p* = 0.045; PR+: *β*_adj._ = − 0.225, *p* = 0.049).

High intake levels of animal proteins (*β*_adj._ = 0.238, *p* = 0.041), as well as the ratios of TP:DF (*β*_adj._ = 0.242, *p* = 0.040) and AP:PP (*β*_adj._ = 0.273, *p* = 0.022), displayed significantly positive correlations with *RhoA* expression, whenever ALNM+ was selected (Table 5). Also, protein provided by red meat (*β*_adj._ = 0.230, *p* = 0.039) and dairy products (*β*_adj._ = 0.271, *p* = 0.030) positively correlated with the *RhoA* expression in ALNM+ status. High protein intake from nuts and cereals was inversely associated with *RhoA* expression in ALNM+ patients (*β*_adj._ = − 0.239, *p* = 0.043). Animal proteins (*β*_adj._ = 0.313, *p* = 0.007), red meat protein (*β*_adj._ = 0.358, *p* = 0.001), and protein from dairy products (*β*_adj._ = 0.275, *p* = 0.035) positively correlated with *RhoA* expression in subjects who had pathologic diagnosis of VI+. High protein intake from nuts and cereals (*β*_adj._ = − 0.234, *p* = 0.045) was inversely associated with *RhoA* expression in VI+ status (Table 6).

#### VEGF-A

High total protein (*β*_adj._ = 0.200, *p* = 0.029), residual total protein (*β*_adj._ = 0.206, *p* = 0.029), and red meat protein (*β*_adj._ = 0.262, *p* = 0.001) correlated significantly with *VEGF-A* expression (Table 2). The overexpression of *VEGF-A* correlated with proteins including processed red meat (*β*_adj._ = 0.184, *p* = 0.043), seafood (*β*_adj._ = 0.180, *p* = 0.038), and dairy products (*β*_adj._ = 0.183, *p* = 0.030) as well.

The correlations of red meat protein (*β*_adj._ = 0.283, *p* = 0.002) and residual total protein intake (*β*_adj._ = 0.311, *p* = 0.004) with fold changes in the expression of *VEGF-A* were observed in ER+ BC patients (Table 3). Similarly, red meat protein (*β*_adj._ = 0.271, *p* = 0.004), total protein (*β*_adj._ = 0.296, *p* = 0.005), and its residual variable (*β*_adj._ = 0.336, *p* = 0.002) positively correlated with *VEGF-A* expression in PR+ BC patients (Table 4).

In ALNM+ patients, only red meat intake was in association with *VEGF-A* expression at crude (*β* = 0.294, *p* = 0.005) and adjusted (*β*_adj._ = 0.321, *p* = 0.003) models (Table 5). On the other hand, subpopulation with ALNM− showed a positive correlation between total protein and upregulation of *VEGF-A* (*β*_adj._ = 0.339, *p* = 0.019). In patients with VI+ (Table 6), there was a positive correlation between red meat protein and *VEGF-A* expression (*β*_adj._ = 0.346, *p* = 0.002). Similarly, the high dairy protein could associate with the overexpression of *VEGF-A* (*β*_adj._ = 0.263, *p* = 0.020) in VI+ status.

#### VEGFR2

High intake of total protein (*β*_adj._ = 0.217, *p* = 0.013) and animal proteins (*β*_adj._ = 0.237, *p* = 0.007) as well as protein provided by red meat (*β*_adj._ = 0.304, *p* < 0.001) and processed meats (*β*_adj._ = 0.191, *p* = 0.039) correlated significantly with the overexpression of VEGFR2 gene (Table 2). A greater ratio of AP: PP (*β*_adj._ = 0.283, *p* = 0.001) was associated with the overexpressed VEGFR2.

Animal proteins (ER+: *β*_adj._ = 0.299, *p* = 0.003; PR+: *β*_adj._ = 0.296, *p* = 0.004), red meat protein (ER+: *β*_adj._ = 0.315, *p* = 0.001; PR+: *β*_adj._ = 0.315, *p* = 0.001), and the ratio of AP:PP (ER+: *β*_adj._ = 0.331, *p* < 0.001; PR+: *β*_adj._ = 0.331, *p* = 0.001) positively correlated with fold changes in the expression of *VEGFR2* (Tables 3 and 4).

Total protein was positively correlated with *VEGFR2* expression (*β*_adj._ = 0.295, *p* = 0.009) in ALNM+ BC patients (Table 5). There was also a positive correlation between protein from processed red meat and *VEGFR2* expression (*β*_adj._ = 0.265, *p* = 0.030). The ratios of TP:DF (*β*_adj._ = 0.305, *p* = 0.010) and AP:PP (*β*_adj._ = 0.290, *p* = 0.018) were the variables significantly correlated with fold change in the expression levels of *VEGFR2* at subgroup of VI+ (Table 6). In patients with VI+, red meat intake was strongly correlated with *VEGFR2* expression (*β*_adj._ = 0.467, *p* < 0.001).

### PCA-based dietary patterns of proteins

By performing PCA analyses, three important dietary protein patterns generated as (1) “whole meat,” (2) “legume dairy products,” and (3) “plant proteins” whereby they all three could explain 49.5% of the total variances accounted for (Additional file 1: Table S1). The *χ*^2^ for Bartlett’s test of sphericity was statistically significant at *p* < 0.001, and the Kaiser-Meyer-Olkin measure of sampling adequacy showed a score of 0.521.

The mean intake of nutrients was compared among each component (protein pattern) stratified by tertile and summarized in Additional file 2: Table S2. Individuals at the highest adherence to the “whole meat” pattern consumed higher intake levels of saturated fat (*p* = 0.015) and cholesterol (*p* = 0.030). The highest tertile of “legume dairy products” pattern consumed higher intake of energy (*p* = 0.008), dietary fiber (*p* < 0.001), insoluble fiber (*p* = 0.015), crude fiber (*p* < 0.001), iron (*p* = 0.001), and folate (*p* < 0.001) than the intake amounts observed in the lowest tertile. Women in the highest tertile of “plants” pattern had less cholesterol intake than the lowest tertile (*p* = 0.008).

The first pattern including protein intakes from seafood, poultries, red meats, and processed meats, in terms of “whole meat,” was inversely associated with *RhoA* expression in ALNM+ (*β*_adj._ = − 0.253, *p* = 0.033) and positively correlated with *VEGFR2* expression in VI+ patients (*β*_adj._ = 0.288, *p* = 0.016). Logistic regression analysis showed that the second quartile of “whole meat” pattern appeared to associate inversely with fold changes in the expression of studied genes (OR_*RhoA*_ = 0.24, 95% CI 0.07–0.83; OR_*VEGF-A*_ = 0.26, 95% CI 0.07–0.97; OR_*VEGFR2*_ *=* 0.27, 95% CI 0.08–0.96). However, the trend of ORs was not significant even after the adjustments made for covariates.

The second pattern including protein intakes from milk, dairy products, and legumes was positively associated with fold changes in the expression levels of *RhoA* (*β*_adj._ = 0.249, *p* = 0.031) and *VEGF-A* (*β*_adj._ = 0.297, *p* = 0.019) in BC patients with VI+. This pattern also correlated with overexpressed *VEGF-A* of those patients classified as ALNM+ (*β*_adj._ = 0.330, *p* = 0.013). No significant association was observed between the plant protein pattern (protein intakes from fruits, vegetables, soybean, potato, cereals, nuts, and seeds) and fold change of the expressions of interested genes.

**Fig. 1:**
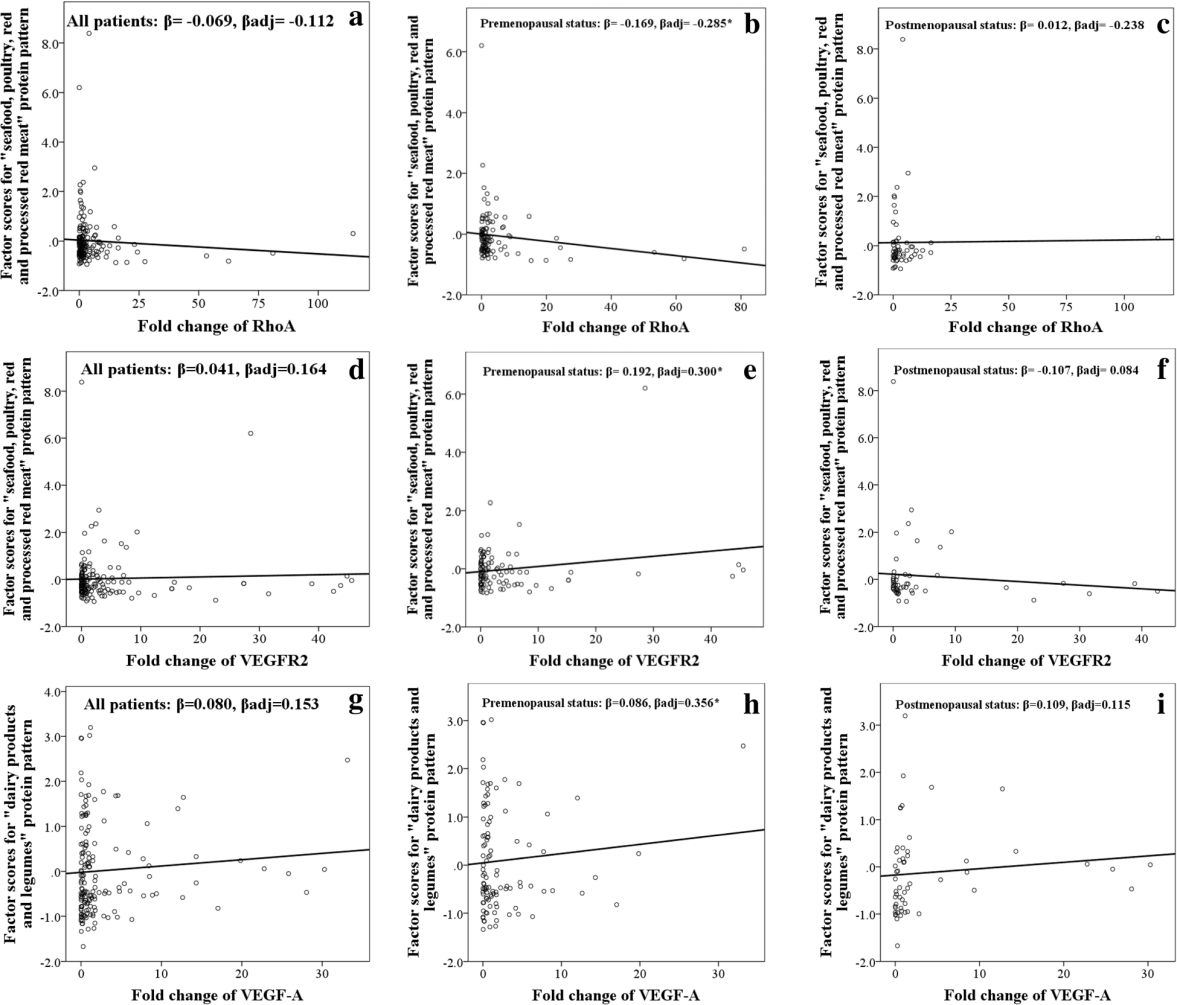
Scatter plots showing linear regression coefficients (standardized *β*) between dietary patterns of protein and studied genes in menopausal status (*N* = 177). **a**–**c** Adjusted for fat (g/day), cholesterol (mg/day), and mean duration of breastfeeding (months). **d**–**f** Adjusted for saturated fat (g/day), tumor size (cm), and waist to hip ratio. **g**–**i** Adjusted for crude fiber (g/day), folate (μg/day), mean duration of breastfeeding (months), and number of abortion

The correlation between dietary patterns of protein and studied genes in menopausal status are shown in Fig. 1. In premenopausal status, the “whole meat” as the first pattern was associated inversely with *RhoA* (*β*_adj._ = − 0.285, *p* = 0.014) and positively with *VEGFR2* (*β*_adj._ = 0.300, *p* = 0.009). Protein provided by “legume dairy products” as the second pattern significantly correlated with the overexpression of *VEGF-A* in premenopausal women (*β*_adj._ = 0.356, *p* = 0.029).

## Discussion

Findings of the present molecular epidemiologic study provided supports in associations between high consumption of protein and upregulation of *RhoA* and *VEGF*-*VEGFR2*. These candidate genes are functionally significant in lymphangiogenesis as a poor determinant of prognosis [23, 24, 27]. Two sets of results were mainly developed. First, proteins from red meat and dairy products were demonstrated to have a significant correlation with the overexpression of *RhoA* in favor of growing tumor cells to lymph nodes (ALNM) and VI+ patients. Similarly, in both clinical subgroups, red meat correlated with upregulated *VEGF-A* and *VEGFR2* to promote ALNM and VI. Secondly, the upstream regulatory effects of ER and PR signaling seem to be a crucial modifier in specifying what type of dietary protein can modify the transcription levels of study gene.

To our knowledge, this is the first study to investigate the association between dietary protein intake and the expression of *RhoA*, *VEGF-A*, and *VEGFR2*. A considerable number of prospective cohort-based studies showed that dietary protein can associate with substantially increased BC risk [7, 8, 37]. Cho and co-workers [7], in a large prospective cohort study, reported that red meat intake strongly elevates BC risk. Lately, Wu et al. [6] conducted a dose-response meta-analysis of prospective studies and revealed that protein obtained by red and processed meat may enhance BC risk. In another cohort study, total red meat intake was positively associated with increased risk of metastasis of breast tumor [38]. Furthermore, in Sweden, Larsson et al. [37] suggested that fried meat intake may enhance the risk of ER+/PR− breast carcinogenesis. By contrast, no population-based study exists showing how dietary protein can affect the molecular responses in metastasis of BC tumors [17].

The overexpression of *RhoA* is the most common feature of metastasis which is unraveled in association with modifiable dietary risk factors [14, 16]. Present findings showed that animal proteins, especially red meat, can increase strongly the expression levels of *RhoA*. This result was also re-emphasized particularly in ER+ and PR+ BCs, showing the possible interfering impacts of active ER and PR signaling over this nutrigenomic model. In detail, plant sources of protein specifically from nuts and cereals correlated inversely with *RhoA* expression varied dramatically by ER+ and PR+ statuses. Several different mechanisms have been proposed to figure out why red meat consumption causes susceptibility to cancer development. Carcinogenic heterocyclic amines (HCAs) formed in meat are contingent with the type of meat (red, white) and the factors associated with the cooking process such as temperature and duration [39, 40]. In vitro experiments showed that specific HCAs, involved 2-amino-1-methyl-6-phenylimidazo[4,5-b]pyridine (PhIP), are a byproduct component with estrogenic effects that could induce a mitogenic response through ER signaling [39, 40]. Moreover, PhIP could also induce upregulation of PR in MCF-7 cells [40]. High intake of animal proteins could increase the acid load in the circulation [18], thereby promoting the formation of stress fiber mediated by *RhoA* in enhancing focal adhesion mechanism [18]. Oster and co-workers [41] undertook microarray analyses on skeletal muscle tissue of 253 offspring of German gilts fed with iso-caloric diets showed that the transcriptional pathway of *Rho* GTPase decreased in “low-protein/high-carbohydrate” diet after 188 days follow-up at postnatal subjects. Garcia et al. [42] indicated that treatment with arachidonic acid in MDA-MB-435 human melanoma cells can activate *RhoA* promoting cell adhesion via p38 MAPK-*RhoA* signaling pathway. Arachidonic acid is rich in animal protein, especially red meat. Thromboxane A2 is a pro-inflammatory factor produced from arachidonic acid and potently participates in platelet aggregation and vascular contraction [42]. Thromboxane A2 is a potent enhancer of *RhoA* transcription [43]. Red meat is rich in *N*-glycolylneuraminic acid (Neu5Gc). Samraj et al. [44] indicated induced hepatocellular tumorigenesis by Neu5Gc acid intervention in human-like Neu5Gc-deficient mice giving support to epidemiologic findings showing the correlation of red meat consumption and risk of cancer incidence. Over an interventional clinical trial-manipulated high intake of red meat in the diet of patients with irritable bowel disease, the expression of *RhoA* increased in colon tissue [17]. Although different aspects exist to support the carcinogenic effects of high protein consumption particularly from animal red meat, our findings can provide new insight indicating significant alterations of *RhoA* expression levels in association with independent dietary factors such as red meat and dairy products.

Animal proteins especially red meat and dairy products were correlated with *RhoA* overexpression dependent on the involvement of ALNM and VI positivity, supported our hypothesis expressed the triple axis of diet, *RhoA* transcription and lymphatic anomalies in BC participants. Although, based on PCA outputs, “whole meat” was associated inversely with *RhoA* expression in ALNM+ and premenopausal status. It is noteworthy to mention that at the present study, white meat (poultries and seafood) was consumed 70% more than red and processed meat. This would explain why the extent of correlation by every individual food item could be hindered or even masked by other prominent dietary counterparts in the model defining the component. Given that very limited information exist to discuss how dietary factors can contribute in the infiltration of tumor cells to the lymph nodes and vessels, epidemiologic studies are widely warranted to study the correlation of protein sources and other metastatic transcripts in future researches.

At the present study, protein from the group of foods including nuts and cereals were inversely associated with *RhoA* expression levels, particularly in ER+ and PR+ subjects and involvement of ALNM and VI positivity, suggesting the possible preventive correlation of nuts and cereals on *RhoA* overexpression. Similarly, in a large population-based case control study, Liu et al. [45] reported that nuts and vegetable proteins in adolescence may associate with reduced risk of BC later in life. However, a meta-analysis of prospective cohort studies has found no linear correlation between nut intake and BC risk [6]. Our results are not consistent with those of a prospective cohort study conducted by Farvid et al. [8] showing that replacing legumes and nuts instead of red meat in early adulthood could reduce BC risk later in life. The nutrigenomic aspect of consuming proteins could be better represented when the planning of models is accounted for controlling the hormonal receptor status of tumors.

It is well established that *VEGF-A* takes part as a potent angiogenic growth factor in the nurturing malignant solid tumors mainly through binding to *VEGFR2* [21]. Molecular evidence indicated that co-expression of *VEGF-A* and *VEGFR2* can associate with poor prognosis and worse clinical outcomes in BC patients [27]. Present results revealed that protein intake from red meat can increase transcript levels of both *VEGF-A* and *VEGFR2* whereby ER and PR were expressed positively. The Western diet including in particular high red meat increases the serum concentrations of free estradiol thereby promoting BC risk [46]. However, little is known about the effects of protein quantity and quality in regulating the molecular pathways which control carcinogenesis. The IGF-1/mTOR signaling pathway is principally regulated by protein content [10, 26]. Studies suggested that dietary protein restriction would be more effective rather than putting a restriction on calories or fat intake in order to decrease IGF-1 levels by inhibiting the PI3K/AKT/mTOR pathway [9, 10, 47]. In an experimental study, mice fed a low-protein diet showed 45% smaller tumor size and 30% less serum concentration of IGF-1 than high protein consumers [10]. In a xenograft model of prostate cancer, mice fed low-protein diet (7% of total calorie) represented a significant decrease in expression of *enhancer of zeste homolog 2* [9] which could enhance *VEGF-A* expression [48]. Isocaloric diet in animal consumed plant proteins significantly inhibit BC growth in human xenograft models of tumorigenesis seems to be mediated by the reduction in serum IGF-1 levels and downregulation of intratumor mTOR activity [9]. Porcine hepatocytes and HepG2 cell line exposed to 4× amino acid concentration showed increased expressions of IGF-1, *peroxisome proliferator-activated receptor* *γ* (*PPARγ*), and *activated protein-2* (*ap-2*) are also significantly overexpressed [49]. Downregulation of *ap-2* can lead to the inhibition of *VEGF* expression in human H1299 cell line [50]. High transcript levels of *PPARγ* could stimulate angiogenesis in various carcinoma through increasing *VEGF* expression [51]. Our findings showed that protein obtained from red meat correlated with upregulated *VEGF-A* and processed meat to higher levels of *VEGFR2* in association with the feature of ALNM+. The overexpression of *VEGF-A* and *VEGFR2* were attributed to high protein intake from red meat when patients’ tumor accounted for the involvement of VI. Moreover, based on PCA data, the protein obtained from “whole meat” was positively associated with *VEGFR2* expression in VI+ patients and premenopausal status. No previous published data is available to compromise how protein does associate with lymphangiogenesis dependent on alteration in genomic profile (such as *VEGFR2*). Given the association of protein intake with overexpression of *VEGF* and *VEGFR2* suggest that this nutrigenomic model can correlate with determining the predispose population to spread tumor cells into lymph node and vessels as important clinicopathologic variables.

The results of this study showed that proteins obtained from legumes (beans, peas, and lentil) and dairy products correlated positively with the increased fold change in the expression of *VEGF-A* at premenopausal status or who characterized by ALNM+ and VI+. While nutraceutical effects of legume consumption raise the issue of possible anti-carcinogenic effects by active ingredients assigned to legumes [52], some epidemiologic studies were unsuccessful to reveal associations between legume intake and cancers of the breast [53], prostate [54], and colon [55]. Our findings showed the positive correlation between “legume dairy products” pattern and *VEGF-A* expressions while it is noteworthy to highlight that the association of legumes might be hampered by the significant correlation of dairy products to the variances displayed by component 2 (legume dairy products). The PCA-independent data was also reassured that, despite legumes, just the group of dairy products was correlated with the upregulation of *VEGF-A*. The nature of milk proteins may explain the positive association between dairy products and the overexpression of *VEGF-A*. Cow’s milk contains two important glycoproteins, lactadherin, and angiogenin-2, which they can modulate angiogenesis process [56]. Lactadherin which is secreted into milk promotes *VEGF*-dependent Akt phosphorylation consequently induces neovascularization [56]. Moreover, hormone-containing cow’s milk may predispose milk consumers to increased IGF-1 and estrogen in time-dependent condition linked as a potent risk factor for BC [57]. The incidence of mammary tumor in rats which exposed to methylnitrosourea and fed a casein-based diet was 80% higher than rats fed with a soy protein diet (42% incidence rate) [58]. By contrast, a recent meta-analysis indicates that increased consumption of total dairy food, but not milk, may associate with a reduced risk of BC [59]. Higher intake of legumes and dairy products may associate with high estradiol levels and increased risk of BC in postmenopausal women [60]. Our findings can provide new insight specifying significant alterations of *VEGF-A* and *VEGFR2* expression levels in association with legumes and dairy products.

This study had some limitations. Aside from the possibility of recall bias which cannot be completely excluded, the sample size was small. A larger population study would be desirable in order to perform the molecular epidemiologic study.

In conclusion, the findings suggested that high intake of animal proteins especially red meat may associate with the overexpression of *RhoA* and *VEGF-VEGFR2* in patients characterized by the involvement of ALNM and VI. Wherein the combination of legume dairy products correlated with *RhoA* and *VEGF-A*, either a clinicopathologic feature of lymphatic or vascular metastasis was remarkable in BC patients. Less intake of “whole meat” was associated with less fold change in the expression of interested genes and may suggest the prevention of metastasis in BC patients. Thus, for future studies, it is highly recommended to study the association between different dietary sources of protein and a larger genomic profile including various metastatic and angiogenic genes.

## Additional files

Additional file 1: **Table S1.** Factor loadings for identified protein patterns (*N* = 177). (DOCX 13 kb)

Additional file 2: **Table S2.** Dietary characteristics across tertile (*T*) of three identified protein patterns (*N* = 172). (DOCX 20 kb)

## Data Availability

The data that support the findings of this study are available from TBZMED, but restrictions apply to the availability of these data, which were used under license for the current study, and so are not publicly available. However, data are available from the authors upon reasonable request and with permission from TBZMED.
