# Supplementary material for: Dietary protein sources and tumoral overexpression of RhoA, VEGF-A and VEGFR2 genes among breast cancer patients
Source: Genes Nutr. 2019 Jul 9;14:22. doi: 10.1186/s12263-019-0645-7 (PMC6617685; doi:10.1186/s12263-019-0645-7)
Supplement: Supplementary file 1 — Table S1. Factor loadings for identified protein patterns (N = 177). (DOCX 13 kb) [file 12263_2019_645_MOESM1_ESM.docx]

| **Supplement Table 1:** Factor loadings for identified protein patterns (N=177) | | | |
| --- | --- | --- | --- |
| **Protein groups** | **Component 1*** | **Component 2** | **Component 3** |
| Protein intake of Seafood | 0.510 |  |  |
| Protein intake of Poultry | 0.769 |  |  |
| Protein intake of Red and processed meat | 0.631 |  |  |
| Protein intake of Dairy products |  | 0.798 |  |
| Protein intake of Legumes |  | 0.755 |  |
| Protein intake of Fruits, Vegetables, Soybean, Potato, and Cereals |  |  | 0.703 |
| Protein intake of Nuts and Seeds |  |  | -0.588 |
| **Variance explained (%)** | **19.62** | **16.03** | **13.93** |
| * Exploratory factor analysis using the Factor procedure. Factor loading <0.5 in absolute values were suppressed. | | | |
