# Supplementary material for: Dietary protein sources and tumoral overexpression of RhoA, VEGF-A and VEGFR2 genes among breast cancer patients
Source: Genes Nutr. 2019 Jul 9;14:22. doi: 10.1186/s12263-019-0645-7 (PMC6617685; doi:10.1186/s12263-019-0645-7)
Supplement: Supplementary file 2 — Table S2. Dietary characteristics across tertile (T) of three identified protein patterns (N = 172). (DOCX 20 kb) [file 12263_2019_645_MOESM2_ESM.docx]

| **Supplement Table 2:** Dietary characteristics across tertile (T) of three identified protein patterns (N=172). | | | | | | | | | | | | | |
| --- | --- | --- | --- | --- | --- | --- | --- | --- | --- | --- | --- | --- | --- |
| **Nutrients** | **Component 1** | | | | | **Component 2** | | | | **Component 3** | | | |
|  |  | **T1** | **T2** | **T3** | ***P*-value*** | **T1** | **T2** | **T3** | ***P*-value** | **T1** | **T2** | **T3** | ***P*-value** |
| **Energy (Kcal/d)** | **Mean** | 2669 | 2695 | 2943 |  | 2514 | 2808 | 2996 |  | 2827 | 2677 | 2799 |  |
|  | **SD** | 942 | 783 | 725 |  | 828 | 826 | 757 |  | 933 | 781 | 751 |  |
|  | **N= 166** | 54 | 57 | 55 | 0.158 | 56 | 57 | 53 | **0.008** | 57 | 54 | 55 | 0.602 |
|  |  |  |  |  |  |  |  |  |  |  |  |  |  |
| **Carbohydrate (g/d)** | **Mean** | 383 | 318 | 346 |  | 350 | 325 | 373 |  | 313 | 385 | 351 |  |
|  | **SD** | 524 | 97.98 | 130 |  | 520 | 88.27 | 137 |  | 111 | 526 | 126 |  |
|  | **N= 170** | 56 | 57 | 57 | 0.548 | 57 | 57 | 56 | 0.718 | 58 | 55 | 57 | 0.475 |
|  |  |  |  |  |  |  |  |  |  |  |  |  |  |
| **Protein (g/d)** | **Mean** | 115 | 120 | 134 |  | 122 | 116 | 131 |  | 133 | 118 | 118 |  |
|  | **SD** | 92.81 | 51.03 | 62.90 |  | 99.24 | 55 | 47.90 |  | 98.15 | 55.48 | 50.18 |  |
|  | **N= 168** | 56 | 56 | 56 | 0.355 | 56 | 57 | 55 | 0.556 | 56 | 55 | 57 | 0.451 |
|  |  |  |  |  |  |  |  |  |  |  |  |  |  |
| **Fat (g/d)** | **Mean** | 82.96 | 83.39 | 96.73 |  | 83.47 | 81.59 | 98.25 |  | 92.05 | 78.70 | 91.99 |  |
|  | **SD** | 47.90 | 41.59 | 59.17 |  | 43.29 | 53.95 | 52.20 |  | 50.49 | 49.02 | 50.80 |  |
|  | **N= 167** | 55 | 56 | 56 | 0.259 | 57 | 55 | 55 | 0.162 | 58 | 54 | 55 | 0.278 |
|  |  |  |  |  |  |  |  |  |  |  |  |  |  |
| **Saturated fat (g/d)** | **Mean** | 26.22 | 26.05 | 33.06 |  | 24.90 | 28.97 | 31.37 |  | 29.04 | 27.90 | 28.33 |  |
|  | **SD** | 14.13 | 11.90 | 17.13 |  | 11.68 | 13.22 | 18.20 |  | 14.42 | 12.56 | 17.30 |  |
|  | **N= 172** | 57 | 58 | 57 | **0.015** | 57 | 57 | 58 | 0.060 | 59 | 56 | 57 | 0.918 |
|  |  |  |  |  |  |  |  |  |  |  |  |  |  |
| **Cholesterol (mg/d)** | **Mean** | 323 | 374 | 455 |  | 396 | 351 | 405 |  | 467 | 363 | 319 |  |
|  | **SD** | 281 | 198 | 302 |  | 311 | 193 | 287 |  | 370 | 192 | 170 |  |
|  | **N= 172** | 57 | 58 | 57 | **0.030** | 57 | 57 | 58 | 0.520 | 59 | 56 | 57 | **0.008** |
|  |  |  |  |  |  |  |  |  |  |  |  |  |  |
| **Dietary fiber (g/d)** | **Mean** | 24.57 | 23.22 | 24.15 |  | 20.22 | 23.89 | 27.89 |  | 24.85 | 22.69 | 24.32 |  |
|  | **SD** | 11.18 | 7.73 | 9.29 |  | 7.63 | 8.16 | 10.84 |  | 10.58 | 8.36 | 9.24 |  |
|  | **N= 167** | 55 | 56 | 56 | 0.746 | 56 | 56 | 55 | **<0.001** | 58 | 54 | 55 | 0.460 |
|  |  |  |  |  |  |  |  |  |  |  |  |  |  |
| **Insoluble fiber (g/d)** | **Mean** | 2.36 | 2.60 | 2.61 |  | 2.11 | 2.48 | 3.01 |  | 2.59 | 2.40 | 2.58 |  |
|  | **SD** | 1.77 | 1.72 | 1.47 |  | 1.18 | 1.79 | 1.83 |  | 1.41 | 1.51 | 2.01 |  |
|  | **N= 168** | 56 | 57 | 55 | 0.672 | 57 | 56 | 55 | **0.015** | 57 | 54 | 57 | 0.807 |
|  |  |  |  |  |  |  |  |  |  |  |  |  |  |
| **Soluble fiber (g/d)** | **Mean** | 0.395 | 0.455 | 0.407 |  | 0.400 | 0.403 | 0.460 |  | 0.447 | 0.393 | 0.417 |  |
|  | **SD** | 0.209 | 0.229 | 0.207 |  | 0.203 | 0.218 | 0.225 |  | 0.215 | 0.163 | 0.256 |  |
|  | **N= 162** | 54 | 56 | 52 | 0.308 | 57 | 54 | 51 | 0.279 | 55 | 52 | 55 | 0.436 |
|  |  |  |  |  |  |  |  |  |  |  |  |  |  |
| **Crude fiber (g/d)** | **Mean** | 9.23 | 8.77 | 9.28 |  | 7.54 | 8.97 | 10.77 |  | 9.92 | 8.26 | 9.03 |  |
|  | **SD** | 4.11 | 3.39 | 4.01 |  | 3.18 | 3.32 | 4.25 |  | 4.08 | 3.00 | 4.14 |  |
|  | **N= 169** | 55 | 57 | 57 | 0.745 | 56 | 57 | 56 | **<0.001** | 58 | 54 | 57 | 0.070 |
|  |  |  |  |  |  |  |  |  |  |  |  |  |  |
| **Iron (mg/d)** | **Mean** | 19.65 | 20.53 | 22.38 |  | 18.52 | 20.18 | 23.82 |  | 21.26 | 19.80 | 21.48 |  |
|  | **SD** | 8.74 | 6.44 | 8.67 |  | 8.33 | 6.96 | 7.98 |  | 8.28 | 8.43 | 7.45 |  |
|  | **N= 172** | 57 | 58 | 57 | 0.183 | 57 | 57 | 58 | **0.001** | 59 | 56 | 57 | 0.485 |
|  |  |  |  |  |  |  |  |  |  |  |  |  |  |
| **Folate (µg/d)** | **Mean** | 396 | 394 | 405 |  | 340 | 370 | 483 |  | 438 | 378 | 377 |  |
|  | **SD** | 172 | 173 | 159 |  | 168 | 135 | 164 |  | 171 | 142 | 181 |  |
|  | **N= 172** | 57 | 58 | 57 | 0.934 | 57 | 57 | 58 | **<0.001** | 59 | 56 | 57 | 0.077 |
| * One-way ANOVA test was performed. | | | | | | | | | | | | | |
